# Phage selection drives resistance–virulence trade-offs in *Ralstonia solanacearum* plant-pathogenic bacterium irrespective of the growth temperature

**DOI:** 10.1093/evlett/qrad056

**Published:** 2023-11-11

**Authors:** Jianing Wang, Xiaofang Wang, Keming Yang, Chunxia Lu, Bryden Fields, Yangchun Xu, Qirong Shen, Zhong Wei, Ville-Petri Friman

**Affiliations:** Key Lab of Organic-Based Fertilizers of China and Jiangsu Provincial Key Lab for Solid Organic Waste Utilization, Nanjing Agricultural University, Nanjing, P.R. China; Key Lab of Organic-Based Fertilizers of China and Jiangsu Provincial Key Lab for Solid Organic Waste Utilization, Nanjing Agricultural University, Nanjing, P.R. China; Key Lab of Organic-Based Fertilizers of China and Jiangsu Provincial Key Lab for Solid Organic Waste Utilization, Nanjing Agricultural University, Nanjing, P.R. China; Key Lab of Organic-Based Fertilizers of China and Jiangsu Provincial Key Lab for Solid Organic Waste Utilization, Nanjing Agricultural University, Nanjing, P.R. China; Department of Microbiology, University of Helsinki, Helsinki, Finland; Key Lab of Organic-Based Fertilizers of China and Jiangsu Provincial Key Lab for Solid Organic Waste Utilization, Nanjing Agricultural University, Nanjing, P.R. China; Key Lab of Organic-Based Fertilizers of China and Jiangsu Provincial Key Lab for Solid Organic Waste Utilization, Nanjing Agricultural University, Nanjing, P.R. China; Key Lab of Organic-Based Fertilizers of China and Jiangsu Provincial Key Lab for Solid Organic Waste Utilization, Nanjing Agricultural University, Nanjing, P.R. China; Key Lab of Organic-Based Fertilizers of China and Jiangsu Provincial Key Lab for Solid Organic Waste Utilization, Nanjing Agricultural University, Nanjing, P.R. China; Department of Microbiology, University of Helsinki, Helsinki, Finland; Department of Biology, University of York, Wentworth Way, York , United Kingdom

**Keywords:** experimental evolution, phage defense systems, phage resistance, small colony variant (SCV), trade-offs, virulence

## Abstract

While temperature has been shown to affect the survival and growth of bacteria and their phage parasites, it is unclear if trade-offs between phage resistance and other bacterial traits depend on the temperature. Here, we experimentally compared the evolution of phage resistance–virulence trade-offs and underlying molecular mechanisms in phytopathogenic *Ralstonia solanacearum* bacterium at 25 °C and 35 °C temperature environments. We found that while phages reduced *R. solanacearum* densities relatively more at 25 °C, no difference in the final level of phage resistance was observed between temperature treatments. Instead, small colony variants (SCVs) with increased growth rate and mutations in the quorum-sensing (QS) signaling receptor gene, *phcS*, evolved in both temperature treatments. Interestingly, SCVs were also phage-resistant and reached higher frequencies in the presence of phages. Evolving phage resistance was costly, resulting in reduced carrying capacity, biofilm formation, and virulence in planta, possibly due to loss of QS-mediated expression of key virulence genes. We also observed mucoid phage-resistant colonies that showed loss of virulence and reduced twitching motility likely due to parallel mutations in prepilin peptidase gene, *pilD*. Moreover, phage-resistant SCVs from 35 °C-phage treatment had parallel mutations in type II secretion system (T2SS) genes (*gspE* and *gspF*). Adsorption assays confirmed the role of *pilD* as a phage receptor, while no loss of adsorption was found with *phcS* or T2SS mutants, indicative of other downstream phage resistance mechanisms. Additional transcriptomic analysis revealed upregulation of CBASS and type I restriction-modification phage defense systems in response to phage exposure, which coincided with reduced expression of motility and virulence-associated genes, including *pilD* and type II and III secretion systems. Together, these results suggest that while phage resistance–virulence trade-offs are not affected by the growth temperature, they could be mediated through both pre- and postinfection phage resistance mechanisms.

## Introduction

*Ralstonia solanacearum* is a notorious soil-borne bacterium, capable of infecting many economically important crops, such as tobacco, potato, and tomato (Jiang et al., 2017). With tomato, *R. solanacearum* causes bacterial wilt disease, blocking water circulation when invading and growing within plant xylem. Unfortunately, no efficient control methods exist, and it is estimated that *R. solanacearum*-inflicted damage to potato alone affects 3.75 million acres in 80 countries with global damage estimates exceeding $950 million per year (Patil et al., 2012). Biological control (biocontrol), the use of antagonists to suppress the growth of plant disease-causing agents, could offer a viable and environmentally friendly solution for crop protection in agriculture (Droby et al., 2009; Lugtenberg & Kamilova, 2009). Especially, obligate viral parasites of bacteria (i.e., bacteriophages, phages for short) have been demonstrated to be effective at controlling *R. solanacearum* population densities in lab and greenhouse experiments, resulting in reduced bacterial wilt disease incidence (Bae et al., 2012; Fujiwara et al., 2011; Wang et al., 2017). Such phage biocontrol is achieved by using lytic phages which always kill (i.e., lyse) bacterial cells to release phage progeny instead of temperate phages, which can also integrate their genome into the host chromosome without killing the host. In addition to regulating pathogen population densities, lytic phages have been shown to select for phage-resistant mutants, which often leads to correlated changes in other pathogen traits, including reduced pathogen virulence and competitiveness due to costly trade-offs (Addy et al., 2012; Wang et al., 2017, 2019). While evolution of phage resistance could reduce the pathogen density control by the phage, phage resistance–host virulence trade-offs could lead to beneficial outcomes if they cause attenuation of disease outbreaks. What remains unclear is how the evolution of resistance–virulence trade-offs is affected by the abiotic environmental factors in *R. solanacearum*, such as temperature. Here we directly tested how the evolution of phage resistance–virulence trade-offs is affected by the environmental temperature, which has previously been shown to affect bacterial wilt outbreaks (Wei et al., 2015, 2017) and the ecology and evolution of phage–bacteria interactions in general (Duncan et al., 2017; Zhang & Buckling, 2011).

Temperature could affect the ecological and evolutionary dynamics of phage–bacteria interactions in multiple ways. For example, high temperature often boosts the growth of *R. solanacearum*, which could result in more severe disease outbreaks in field conditions via more efficient plant colonization (Wei et al., 2015, 2017). While phages are often able to track changes in *R. solanacearum* population densities and exert efficient top-down control on the pathogen (Wang et al., 2019; Yang et al., 2023), this might not always be the case if phages show higher sensitivity to elevated temperature (Persat et al., 2015; Zhang & Buckling, 2011), or fail to coevolve with bacteria (Zhang & Buckling, 2016). Temperature variation has also been shown to increase fluctuations in both bacteria and phage densities (Duncan et al., 2017), which could increase the risk of extinctions over a longer time scale. In addition to ecological dynamics, population density fluctuations will also affect the mutation supply rate and the frequency of phage–bacteria encounters, resulting in variation in the strength of selection for resistance and infectivity in time (evolutionary dynamics) (Duncan et al., 2017). For example, an increase in environmental temperature could select for higher levels of phage resistance if high temperature has positive effects on *R. solanacearum* growth, mutation supply rate and the frequency of phage–bacteria encounters (Duncan et al., 2017). Such escalation in phage resistance could be balanced out by associated costs of resistance. For example, it has been shown that such costs of phage resistance can be highest near the bacterial thermal optimum (Padfield et al., 2020), which could limit phage resistance evolution in environments where bacteria have adapted. Another study demonstrated that the cost of phage resistance increases with the measurement temperature (Quance & Travisano, 2009), which could potentially limit the evolution of resistance along thermal gradients. Finally, growth temperature could modify the expression of bacterial surface proteins, which could prevent phage adhesion to their receptors, affecting phage resistance through phenotypic plasticity (Sillankorva et al., 2004; Tokman et al., 2016). Environmental temperature could hence have complex and nonlinear effects on phage resistance and its associated costs.

Phage resistance could lead to reduced virulence due to the loss of important virulence determinants that have a dual function as phage receptors (Gurney et al., 2020). These structures include, for example, flagella, pilus, LPS, and capsule (Labrie et al., 2010), and mutations in genes encoding these structures often lead to phage resistance and altered virulence (Leon & Bastias, 2015). In addition to phage receptor mutations, bacteria protect themselves against phages using different defense systems, including CRISPR-Cas, Restriction modification (RM), and abortive infection systems amongst others (Doron et al., 2018). While several phage defense systems have been identified in *Ralstonia* (Castillo et al., 2020; Xavier et al., 2019), a lot can yet be described for their role in phage resistance and potential virulence trade-offs. While defense systems can incur costs, these are thought to be relatively smaller compared to receptor mutations as the expression of defense systems are particularly relevant when under phage attack. For example, CRISPR-Cas defense is favored over receptor mutations when the bacterial competition, and costs for carrying receptor mutations, is relatively high (Alseth et al., 2019). The activity of defense systems can also be affected by the temperature. For example, CRISPR-Cas activity has been shown to be boosted by low temperatures via effects on *P. aeruginopsa* growth (Hoyland-Kroghsbo et al., 2018), while elevated temperatures have been shown to abolish the functioning of Dnd R-M system (Jiang et al., 2023). While it has been shown that phages can constrain the evolution of bacterial virulence-associated traits in high-temperature environment (Friman et al., 2011), the effects of temperature on resistance–virulence trade-offs are not yet well understood.

Here we used laboratory experimental evolution to test how environmental temperature (25 °C vs. 35 °C) affects the evolution of phage resistance and associated trade-offs in the *R. solanacearum* phytopathogenic bacterium. Our results show that prior exposure to high temperature was linked to higher phage resistance due to selection for resistant small colony variants (SCVs) irrespective of phage presence. Likely explanation for this was the relatively higher growth rate of SCVs, which increased their competitive fitness relative to ancestral strain. Crucially, phages enriched these resistant SCV mutants in both temperature treatments. Mechanistically, phage resistance was associated with mutations in genes encoding type IV pilus, T2SS and QS-response regulator (*phcS*), resulting in reduced virulence in planta. Moreover, by using transcriptomics, we show that short-term phage exposure leads to expression of RM and CBASS phage defense systems, which coincides with reduced expression of type IV pilus and type II and III secretion system genes associated with virulence. Together, these findings suggest that both pre- and postinfection phage resistance mechanisms could correlate negatively with *R. solanacearum* virulence.

## Methods

### Bacterial strain and phage

*Ralstonia solanacearum* strain RS-N (Wang et al., 2023), isolated in 2016 from the rhizosphere of tomato plant in Qilin, Nanjing, China, was used as the model pathogen strain in all experiments. According to the current species level classification, RS-N belongs to *Ralstonia pseudosolanacearum* species (Safni et al., 2014). However, as the *Ralstonia* species classification is relatively new, this strain is still referred as *R. solanacearum* throughout the text. During the experiments, RS-N was routinely cultured in a liquid nutrient medium (NB; 10.0 g of glucose, 5.0 g of peptone, 0.5 g of yeast extract, 3.0 g of beef extract, 1 L of H_2_O, pH 7.0) at 30 °C. To isolate evolved RS-N colonies, TTC agar plates were used (NB media supplemented with 30 g of agar per liter before autoclaving and addition of 5-ml sterilized 1% 2,3,5-triphenyte-trazoliumchloride solution afterwards). Furthermore, two *R. solanacearum* transposon mutants and their phage susceptible ancestral strains (OE1-1 and QL-Rs1115) were used for testing the effect of individual mutations for the phage resistance. These included *pilB* mutant, which was kindly provided by Yong Zhang, Southwest University, China (unpublished data; OE1-1 background) and *pilM* mutant, which was constructed in our lab in QL-Rs1115 background (Wang et al., 2023) (for method, see Supplementary Table S6). The lytic phage, NNP42, was used as the model phage in all experiments, which was isolated from the rhizosphere of tomato plant in Nanning Guangxi province (China). NNP42 belongs to *Firingavirus* (genus) and is highly infective podophage of RS-N, forming clear plaques on soft agar plates (Wang et al., 2019). To propagate and store the phage for the experiments, RS-N bacterial culture was first grown in 3 ml of NB in six-well plate with shaking 170 rpm at 30 °C until OD_600_ 0.5 (10^7^ CFU/ml) at MOI of 0.1. After 18 hr of growth, bacterial cells were removed by centrifugation (16,000 × g for 3 min), and supernatant filtered through a 0.22-μm membrane filter to collect phage particles, which were stored at 4 °C until later use (Wang et al., 2017).

### *Ralstonia solanacearum*–phage selection experiment

**Figure 1.**
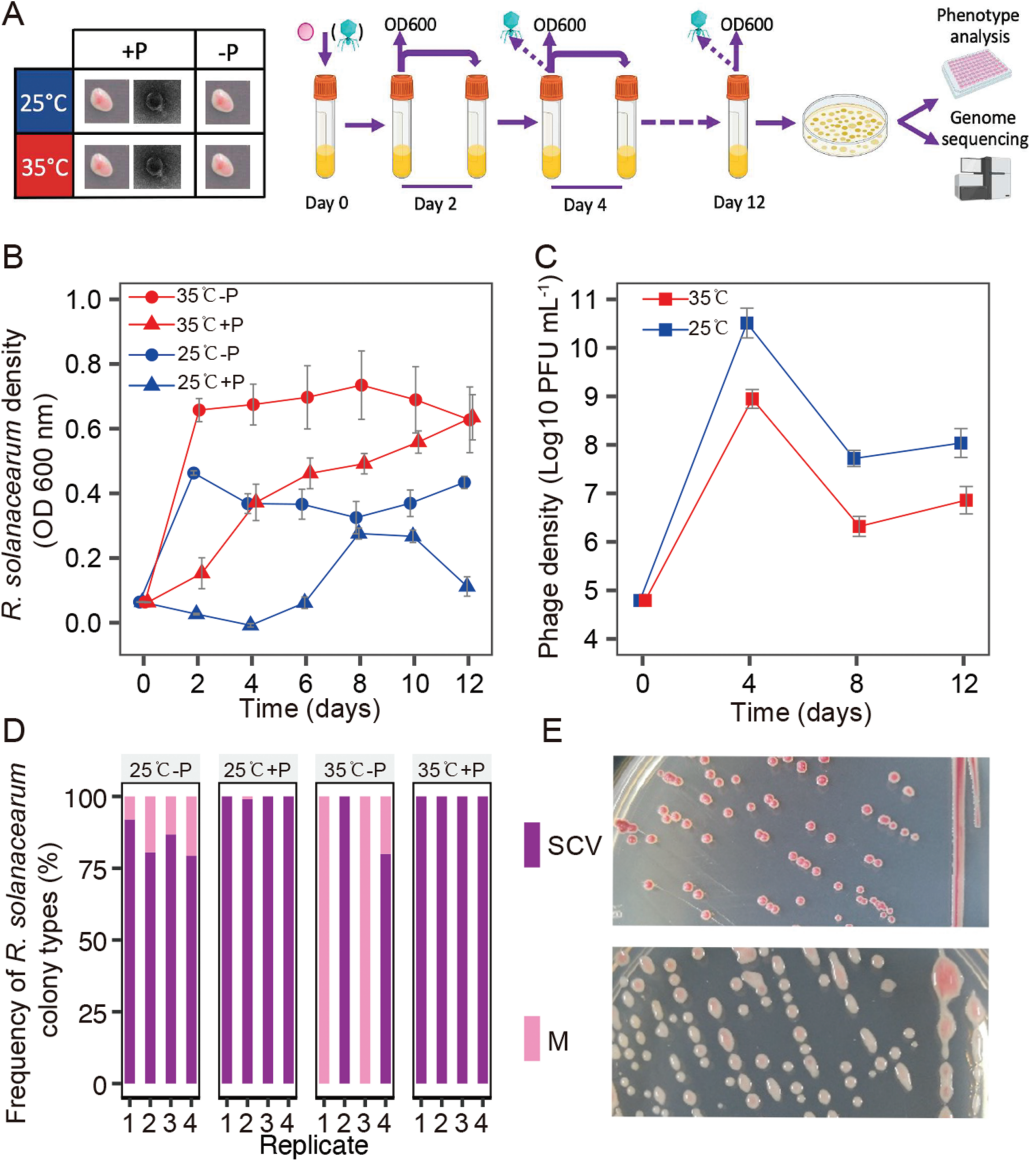
Schematic representation of the experimental design (A), where *Ralstonia solanacearum* RS-N was evolved in the absence and presence of phage NNP42 at 25 or 35 °C for 12 days with 2-day serial transfers. Bacterial and phage densities were recorded at every transfer (2 days) and final time point samples were used to quantify changes in *R. solanacearum* phage resistance, growth and virulence, and to identify parallel mutations between treatment replicates. Population density dynamics of *R. solanacearum* (B) and NNP42 phage (C) during the selection experiment. *N* = 10 for (B) and (C) and bars show the standard error of the mean (± 1 SEM). In (D), stacked histograms show the frequencies of mucoid (pink, “M”) and small colony variants (purple, “SCV”) in each sampled treatment replicate at the Day 12 (*N* = 4). In (B) and (D), the “+P” and “–P” denote for phage presence and absence, respectively. (E) Example morphologies for SCV (top) and mucoid (bottom) colony morphologies on TTC agar plates.

Laboratory experimental evolution approach was used to evolve mucoid ancestral *R. solanacearum* RS-N and select evolved mutants that were resistant to NNP42 phage. To do so, the bacterial cells were cultured in the absence (−P) or in the presence (+P) of phage in 5 ml of NB medium (15-ml falcon tubes with loose lids to ensure aerobic growth conditions) and incubated at low (25 °C) and high (35 °C) temperatures with shaking 170 rpm, respectively (Fig. 1A). All treatments were replicated for 10 times and cultures were established by first inoculating tubes with final density 10^7^ CFU/ml cells of isogenic *R. solanacearum* ancestral bacterium. Phage treatments were further inoculated with a final density 10^4^ PFU/ml particles of isogenic ancestral phage, resulting in MOI of 0.001. All replicate cultures were shaken (170 rpm) and incubated in the corresponding temperature treatments. After 48 hr of growth, evolved replicate populations were homogenized by shaking and 50 μl (1%) of evolved populations were serially transferred to new tubes with 5 ml of fresh NB media. All cultures were propagated for five serial transfers (total of 12 days, estimated 200 bacterial generations). Before every serial transfer, bacterial optical densities (OD_600_) were measured using SpectraMax M5 spectrophotometer (Molecular Devices, Sunnyvale, CA). Phage densities were quantified at every second transfer by plating out phage dilutions onto soft agar plates containing a lawn of ancestral RS-N strain and counting the number of plaque-forming units after 24 hr of incubation (Wang et al., 2019). Subsamples of each replicate were frozen at −80 °C in 30% (v:v) glycerol at every two transfers for later analyses.

### Measuring evolutionary changes in *R. solanacearum* phage resistance

The evolution of *R. solanacearum* phage resistance was quantified at the end of the selection experiment using the last sampled time point (48-h growth after the fifth transfer) to keep the workload manageable and to test phage resistance evolution on a longer time scale typical for field application. We used intermediate, “common garden” temperature of 30 °C to compare the performance of bacterial colonies isolated from low- and high-temperature environments to nullify the prior effects of temperature adaptation on phage resistance-associated traits. RS-N colonies were calculated and isolated from four randomly selected replicates of each treatment using serial dilutions and plating on TTC agar plates. After 48 hr of growth at 30 °C, total colony numbers and frequency of different colony morphotypes were recorded. Eight colonies per replicate population (*N* = 4) were randomly picked, resulting in a total of 32 colonies per treatment. When both types of colonies were observed within the same replicate, four mucoid and four SCVs were randomly isolated per replicate population (Supplementary Figure S1). We also tested the stability of SCV colony types by serially diluting and replating SCV colonies for a second round of growth on NB agar plates. No reversion to mucoid phenotypes was observed, which indicates that SCV colony types were stable at this time scale even when the phage selection pressure was removed.

The phage resistance was determined by culturing evolved *R. solanacearum* isolates in the presence (+) and absence (−) of ancestral phage in liquid medium and determining the relative growth reduction by the phage as optical density (OD_600_) after 24 hr of growth (Wang et al., 2017, 2019; Wright et al., 2018). Phage resistance (R) was quantified as: R = OD_600_(+)/OD_600_(−); low relative density reduction was defined as high phage resistance. Phage resistance measurements were conducted on 96-well microplates in 200 μl of NB. Each well was initially inoculated with approximately 10^6^ CFU/ml of evolved *R. solanacearum* cells, and phage-treatment wells were co-inoculated at the same time with 10^5^ PFU/mL of ancestral phage (MOI of 0.1). All microplates were cultured with shaking at 170 rpm and phage resistance of all evolved, and the ancestral clone was measured in three technical replicates. The same method was used for testing the phage resistance of two transposon mutants (*pilB* and *pilM*) and their wild types.

### Measuring changes in pathogen growth traits

To test whether the evolution of phage resistance was accompanied by growth costs, we measured growth curves for all evolved *R. solanacearum* isolates in NB in the absence of phages. The experiment was conducted on 96-well microplates at 30 °C with shaking 170 rpm as described previously and bacterial growth was measured 0, 4, 8, 12, 20, 24, 28, 36, and 48 hr after initial inoculation. These data points were used to calculated bacterial maximum growth rate and carrying capacity based on logistic models of growth (Wang et al., 2019), indicative of *R. solanacearum* competitive ability (see “Statistical methods” section for more detail). The growth of each isolated clone was measured in three technical replicates.

### Twitching motility assay

To quantify evolutionary changes in *R. solanacearum* twitching motility, plate assays were performed for a subset (*N* = 29) of clones using 1.7 % w/v agar plates. The chosen isolates included four mucoid and four SCV isolates from 25 °C temperature treatments evolved in the absence (−P) or presence of phage (+P). Moreover, four mucoid and four SCV clones were selected from 35 °C temperature treatment evolved in the absence (−P) of phages, while only four SCV clones were isolated from 35 °C temperature treatment evolved in the presence (+P) of phages as no mucoid colonies were observed in this treatment. Additionally, one ancestral clone was included in the assay. Briefly, bacterial isolates were grown overnight in NB at 30 °C, then centrifuged at 1,330 × g for 10 min. Subsequently, bacterial cells were washed twice with sterilized water and resuspended in sterile water (OD _600 nm_ = 1.0) before spot-plating 2 μl of cultures onto the agar plates. Twitching motility was observed by measuring the diameter of the spotted culture for 72 hr based on three technical replicates.

### Biofilm formation assay

For the biofilm formation assay, the same subset of bacterial clones (*N* = 29) were grown in minimal salt glycerol glutamate (MSgg) medium, which was prepared as previously reported (Branda et al., 2001): 5 mM potassium phosphate (pH 7), 100 mM MOPS (pH 7), 0.5% glycerol, 0.5% glutamate, 50 mg/ltryptophan, 50 mg/l phenylalanine, 2 mM MgCl_2_, 0.7 mM CaCl_2_, 50 µM MnCl_2_, 50 µM FeCl_3_, 1 µM ZnCl_2_, and 2 µM thiamin. The biofilm formation was measured using a modified method by O’Toole and Kolter (1998) as follows. First, 10 μl of overnight cultures (in NB) of isolated clones were inoculated to 700 μl of MSgg media on 24-well plate, which was further incubated at 30 °C for 48 hr. After incubation, cultures were discarded, and 700 μl of 1% crystal violet solution was added to each well for biofilm dying at room temperature. After 20 min, crystal violet was discarded, plates rinsed with water five times, and 700 μl of washing buffer (20% acetone and 80% ethanol) added into each well to solubilize the crystal violet. The plate was shaken at 100 rpm for 10 min after 100 μl of solution was pipetted onto 96-well plates, and biofilm formation was determined as optical density at 570 nm. Biofilm formation was measured in three technical replicates.

### Measuring changes in *R. solanacearum* virulence in planta

The same subset of bacterial clones (*N* = 29) was used to quantify *R. solanacearum* virulence in planta. Tomato seeds (cultivar Hongaisheng) were surface sterilized by immersing them in 70% ethanol. After washing with sterile water, seeds were submerged in household bleach (2% NaClO) for 5 min and washed again with sterile water. Seeds were germinated in darkness at 30 °C on water-wetted filter paper for 2 days after germinated seeds were sowed in pots containing 0.6 kg of soil substrate (which is made of mud carbon soil, washed coconut bran, horticultural perlite, horticultural vermiculite, edible dregs, carbonized rice husk, slow-release organic fertilizer, and commercially available from Jiangsu-Xingnong Substrate Technology Co., Ltd). Seedlings were grown in a greenhouse with natural temperature and light variation and watered regularly with sterile water for 6 weeks. The evolved and ancestral clones were grown in NB medium for 2 days at 30 °C. After centrifugation (3,380 × g, 10 min), cells were washed and resuspended in sterile distilled water at a density of 10^8^ CFU/mL (OD_600_ = 0.7), and 10 μl of ancestral and evolved clones’ cell suspensions were injected with a needle into the major stem of 6-week-old tomato plants. As a negative control, 10 μl of sterilized water was injected to control plant stems. Each bacterial clone was tested in three blocks (three replicates) and each block consisted of six tomato plants. After infections, tomato plants were grown in the same conditions in the greenhouse and monitored daily for wilting symptoms. By the end of the experiment (6 weeks postinoculation), the disease incidence was calculated as the percentage of wilted plants (showing any wilt symptoms) per block for comparing treatment means.

### Bacterial DNA extraction and whole-genome-sequencing analysis

The ancestral RS-N strain and evolved strains were grown on NB agar plates at 30 °C for 48 hr, after genomic DNA was isolated from the cell pellets using a Bacteria DNA extraction Kit (OMEGA) following manufacturer’s instructions. Genomic DNA was quantified using TBS-380 fluorometer (Turner BioSystems Inc., Sunnyvale, CA) and DNA shearing was checked using gel electrophoresis. High-quality DNA samples (OD260/280 = 1.8–2.0, >6 µg) were used to construct the fragment library. The RS-N genome was sequenced using a combination of PacBio and Illumina sequencing platforms (Shanghai BIOZERON Co., Ltd), resulting in a complete fully assembled reference genome (Wang et al., 2023). First, the raw paired-end reads obtained by Illumina platform were trimmed and quality controlled by Trimmomatic to obtain clean data. Based on clean data, we used ABySS (http://www.bcgsc.ca/platform/bioinfo/software/abyss) for the genome assembly with multiple-Kmer parameters to attain optimal assembly. Second, canu (https://github.com/marbl/canu) was used to assemble the PacBio corrected long reads. Finally, GapCloser was applied to fill up the remaining local inner gaps and correct the single base polymorphism (https://sourceforge.net/projects/soapdenovo2/files/GapCloser/) for the final assembly. The complete circle of the genome was drawn with Circos v0.64 (http://circos.ca/), and the strain “RS-N” was deposited in GenBank with accession numbers CP099579–CP099581. The Illumina sequenced raw reads for the 28 evolved *R. solanacearum* clones were quality-checked with FastQC (v.0.11.4) (Andrews, 2010). Samples identified to have read files containing an observable percentage of Illumina adapter contamination were removed from adapters using cutadapt (v.2.3) (Martin, 2011). Raw reads for each sample were assembled into contigs de novo with SPAdes (v. 3.14.0) using the isolate mode (Sarovich & Price, 2014). Variants for evolved clones were identified by mapping reads to PacBio reference of *R. solanacearum* strain RS-N using Snippy (v.4.6.0; https://github.com/tseemann/snippy). A GenBank file was created with gene annotations using PROKKA (v.1.14.5; Kingdom: Bacteria, Genus: *Ralstonia*), which was used with Snippy to assign gene information to identified variants (Seemann, 2014). BLASTp was utilized to identify the predicted protein functions of identified hypothetical proteins. The strain “RS-N” was deposited in GenBank with accession numbers CP099579–CP099581.

### Transcriptomic analysis

To evaluate changes in RS-N gene expression in response to phage infection, ancestral RS-N strain was cocultured with the ancestral NNP42 phage at MOI of 0.1 for 6 hr in NB medium in five replicates (or alone as control) at 30 °C with shaking at 170 rpm. This time point was chosen so that enough mRNA transcripts could be collected with no visible emergence of phage-resistant mutants (no increase in bacterial densities in the presence of phage was observed during 24 hr of coculturing, Supplementary Figure S2). The RNA was extracted from 12 ml of cultures and co-cultures compared with RS-N cultured alone for the same amount of time. To minimize RNA degradation, all whole samples were immediately mixed with 3.75 ml of an ice-cold transcriptional stop solution (5% water-saturated phenol in ethanol). Samples were then centrifuged with 10,000 g at 4 °C and pellets were frozen in liquid nitrogen and stored at 80 °C. Total RNA was extracted from the samples using TRIzol Reagent according to the manufacturer’s instructions (Invitrogen) and genomic DNA was removed using DNase I (TaKara). The RNA quality was determined using 2100 Bioanalyser (Agilent) and quantified using the ND-2000 (NanoDrop Technologies). The cDNA synthesis, end repair, A-base addition and ligation of the Illumina-indexed adaptors were performed according to Illumina’s protocol. Libraries were then size selected for cDNA target fragments of 200–300 bp on 2% Low Range Ultra Agarose followed by PCR amplification using Phusion DNA polymerase (NEB) for 15 PCR cycles. After quantification by TBS-380, Paired-end libraries were sequenced by Illumina NovaSeq 6000 sequencing (150 bp × 2, Shanghai BIOZERON Co., Ltd). To process the data, “cutadapt” (Martin, 2011) was used to remove adapters and low-quality sequences at a cutoff of error-rate = 0.1, overlap = 5, minimum-length = 15. Hisat (Kim et al., 2019) was then used to map sequence reads to RS-N PacBio reference genome. KOBAS (http://kobas.cbi.pku.edu.cn/home.do) was used to conduct KEGG pathway enrichment analysis. The HTSeq (Anders et al., 2015) was used to quantify raw expression counts of each gene in each sample, while DESeq2 (Love et al., 2014) was used for differential expression analysis between control and phage treatments. Transcriptomics raw data can be shared on request from the authors.

### Statistical analyses

All time-dependent data were analyzed using repeated-measures ANOVA using populations as subjects and time and other factors as explanatory variables. One- and two-way ANOVA were used for all other analyses without time structure, and pairwise comparisons were confirmed using the Tukey multiple range test. Before analyses, the phage density data was log10-transformed to fulfill the parametric model assumptions. To obtain bacterial growth rates and maximum densities, we used OD_600_ value from 13 time points (0, 4, 8, 12, 20, 24, 28, 36, and 48 hr after initial inoculation) to fit in Logistics growth model function (“gcFitModel” in package “grofit” in R 3.3.1). The slope (μ) and maximum value (*A*) of the growth curve were used to determine the bacterial growth rate and carrying capacity, respectively (Kahm et al., 2010; Yang et al., 2017). All analyses were performed using R version 3.6.3. All statistical tests performed in this study were considered significant at *p* < .05.

## Results

### *Ralstonia solanacearum* and phage population density dynamics

We first compared bacterial and phage population dynamics in two temperatures. In general, *R. solanacearum* reached higher population densities at 35 °C than at 25 °C (temperature, *F*_1,36_ = 73.989, *p* < .001, Supplementary Table S1a, Figure 1B). While the presence of phage significantly reduced *R. solanacearum* densities at both temperatures (phage: *F*_1,36_ = 48.80, *p* < .001, Supplementary Table S1a, Figure 1B), the phage effect on bacteria gradually diminished over time, likely due to the emergence of phage-resistant mutants (temperature × phage × time: *F*_5,180_ = 3.870, *p* < .01, Figure 1B). To compare the strength of phage selection, we calculated the area under the *R. solanacearum* density curves for the whole duration of the experiment and compared the density reduction by phages between two temperature environments. We found that phages reduced the bacterial growth more clearly at 25 °C compared with 35 °C temperature treatment (66.8% vs. 37.18% density reductions; 25 °C: *F*_1,19_ = 88.78, *p* < .001, 35 °C: *F*_1,19_ = 15.66, *p* < .001, respectively). As a result, relatively higher phage densities were found at 25 °C than 35 °C treatment, while phage density dynamics showed similar patterns by rapidly increasing at the beginning of the experiment before reducing over time (temperature: *F*_1,18_ = 32.75, *p* < .001; time: *F*_1,18_ = 84.88, *p* < .001, Supplementary Table S1b, Figure 1C). These results suggest that phage selection was potentially stronger at 25 °C temperature treatment.

We also explored changes in *R. solanacearum* colony morphology frequencies at the final sampling time point by plating randomly chosen four replicates per treatment. We found that ancestral mucoid (M) colony types were lost in the presence of phage at both temperature treatments and replaced by small non-mucoid colony variants (SCV), except for one phage treatment replicates at 25 °C (Figure 1D; hardly visible in the mean plot due to low frequency of SCVs: 0.24% of all colonies within this treatment). While the frequency of SCVs also considerably increased in the absence of phage, around 20% and 55% colonies still had mucoid colony morphology at low- and high-temperature treatments, respectively (phage: *F*_1,12_ = 7.016, *p* < .05, Supplementary Table S2, Figure 1D and E). These results suggest that while culturing conditions alone selected for loss of mucoidy, phage selection enforced this phenotype leading to relatively higher SCV frequencies at both temperature treatments.

### SCVs have relatively higher phage resistance

**Figure 2.**
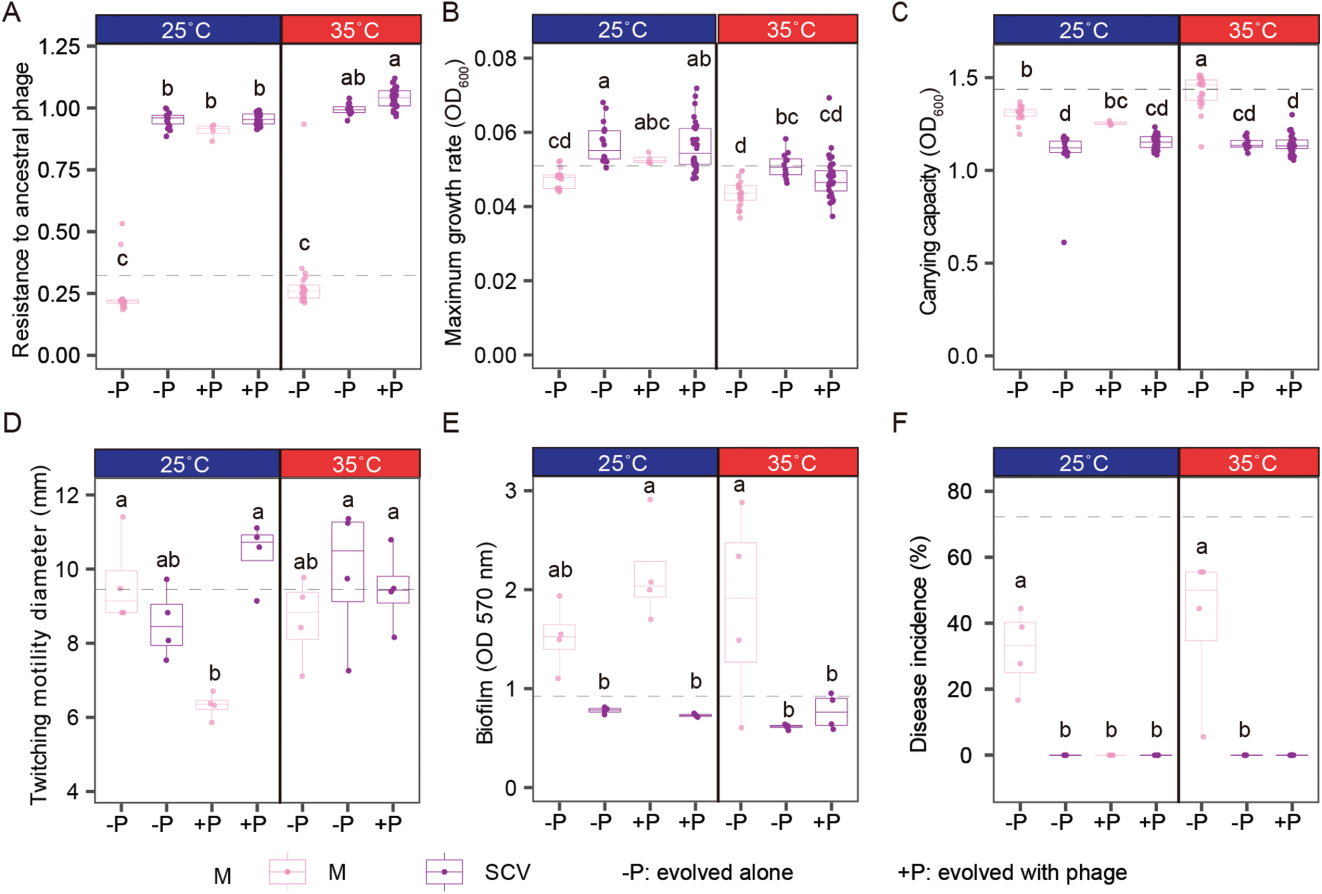
Evolution of phage resistance and correlated changes with growth and virulence in *R. solanacearum* at the end of the selection experiment (day 12). (A–F) show evolved clones’ traits relative to ancestral strain (dashed lines) regarding phage resistance (A), maximum growth rate (B), maximum population density (C), twitching motility (D), biofilm formation (E), and virulence in planta (F), respectively. In (A–F), small letters above boxplots show significances between treatments (*P* < .05), blue and red panel headings denote for prior temperature treatments and M (pink color) and SCV (purple color) denote for mucoid and non-mucoid colonies, respectively. No mucoid colonies were found at 35 °C temperature treatment in the presence of phage. *N* = 4 for all treatments and boxplots show the interquartile range (25%–75% of the data), the median as lines and replicate means as dots (based on eight clones isolated per replicate for A–C and four mucoid clones and four SCVs isolated per treatment for D–F).

To quantify the level of phage resistance, we randomly selected 128 *R. solanacearum* clones with SCV and mucoid colony morphologies from all treatments (eight clones per treatment replicate) at the final time point of the experiment (day 12). We found that prior exposure to high temperature was linked to higher phage resistance (temperature: *F*_1,15_ = 6.459, *p* < .05, Supplementary Table S3a, Figure 2A). However, clones that had previously been exposed to phages showed higher levels of phage resistance compared to no-phage control treatments in both temperature treatments (phage: *F*_1,15_ = 280.752, *p* < .001, Supplementary Table S3a, Figure 2A; phage × temperature: *F*_1,15_ = 2.099, *p* = .168, Supplementary Table S3a, Figure 2A). Interestingly, SCVs were much more resistant to phages compared to mucoid colonies (colony morphology: *F*_1,15_ = 445.207, *p* < .001 Supplementary, Table S3a, Figure 2A) regardless of if they had been exposed to phages during the selection experiment (colony type × phage: *F*_1,15_ = 86.063, *p* < .001, Supplementary Table S3a, Figure 2A). The only exception was mucoid colonies isolated from one treatment replicate at 25 °C in the presence of phage that also showed an increase in phage resistance (phage: *F*_1,18_ = 174.5, *p* < .001). Together, these results suggest that while the level of phage resistance evolution was not affected by the temperature, phage-resistant SCVs that emerged in all treatments were enriched in the presence of phage as selective pressure.

### SCVs show increased growth rate but reduced carrying capacity

To understand the selective benefits of SCVs in the absence of phage, we compared the growth of all evolved *R. solanacearum* clones with the ancestral strain in the absence of phage in a liquid NB medium. We found that SCVs had higher growth rate compared to mucoid colonies (colony type: *F*_1,15_ = 29.393, *p* < .001, Supplementary Table S3b, Figure 2B) regardless of the prior phage exposure (phage × colony type: *F*_1,15_ = 2.898, *p* = .109, Supplementary Table S3b, Figure 2B) or temperature exposure (temperature × colony type: *F*_1,15_ = 0.002, *p* = .967, Supplementary Table S3b, Figure 2B). Interestingly, we saw inverse pattern with carrying capacity: non-mucoid SCVs showed a clear reduction in their carrying capacity (colony type: *F*_1,15_ = 87.965, *p* < .001, Supplementary Table S3c, Figure 2C), which was not affected by prior temperature (temperature × colony type: *F*_1,15_ = 3.222, *p* = .093, Supplementary Table S3c, Figure 2C) and phage exposure treatments (phage × colony type: *F*_1,15_ = 2.900, *p* = .109, Supplementary Table S3c, Figure 2C). These results suggest that SCVs had an increased growth rate, which could explain why they became more prevalent even in the absence of phage.

### Phage resistance and loss of mucoidy leads to reduced *R. solanacearum* virulence

To explore whether SCV morphology and phage resistance correlated with changes in *R. solanacearum* virulence, we quantified changes in twitching motility, biofilm formation and virulence in planta in evolved clones and one ancestral strain (29 clones in total). While prior temperature or phage exposure had no effect on the twitching motility of SCVs (all *p* > .05, Supplementary Table S3d), phage-resistant mucoid colony types from the treatment at 25 °C in the presence of phage showed a clear reduction in twitching motility (*F*_1,6_ = 27.22, *p* < .01, Figure 2D). Prior temperature or phage exposure had no significant effects on biofilm formation (all *p *> .05, Supplementary Table S3e), and overall, mucoid colonies showed increased biofilm formation relative to ancestral strain, while SCVs retained ancestral-level biofilm formation (colony type: *F*_1,21_ = 42.370, *p* < .001, Supplementary Table S3e, Figure 2E). To compare changes in virulence in planta, we conducted a greenhouse experiment where the same evolved and ancestral clones were injected directly into tomato plant stems. We found that prior exposure to the phage resulted in reduced virulence, irrespective of the temperature environment in which the bacteria had evolved (phage: *F*_1,21_ = 20.310, *p* < .001, Supplementary Table S3f, Figure 2F; the main effect of temperature was nonsignificant). Moreover, all SCVs showed complete loss of virulence regardless of if they had previously been exposed to phage (colony type: *F*_1,21_ = 33.413, *p* < .001, Supplementary Table S3f, Figure 2F). Interestingly, the phage-resistant mucoid colonies isolated from 25 °C-phage treatment lost their virulence (colony type × phage: *F*_1,21_ = 9.992, *p* < .01, Supplementary Table S3f, Figure 2F). Together, these results suggest that SCV and phage resistance were associated with a reduction in *R. solanacearum* virulence.

### Genetic changes associated with SCVs and phage resistance

To link SCVs and phage resistance with underlying genetic changes, we sequenced both colony types from all treatments (four clones per treatment, one clone per replicate). Sequenced clones had an average of 8.5 nonsynonymous mutations each, which included small deletions, small insertions, and single-nucleotide polymorphism (Supplementary Table S4). The average number of detected mutations was not affected by the colony morphology.

**Figure. 3:**
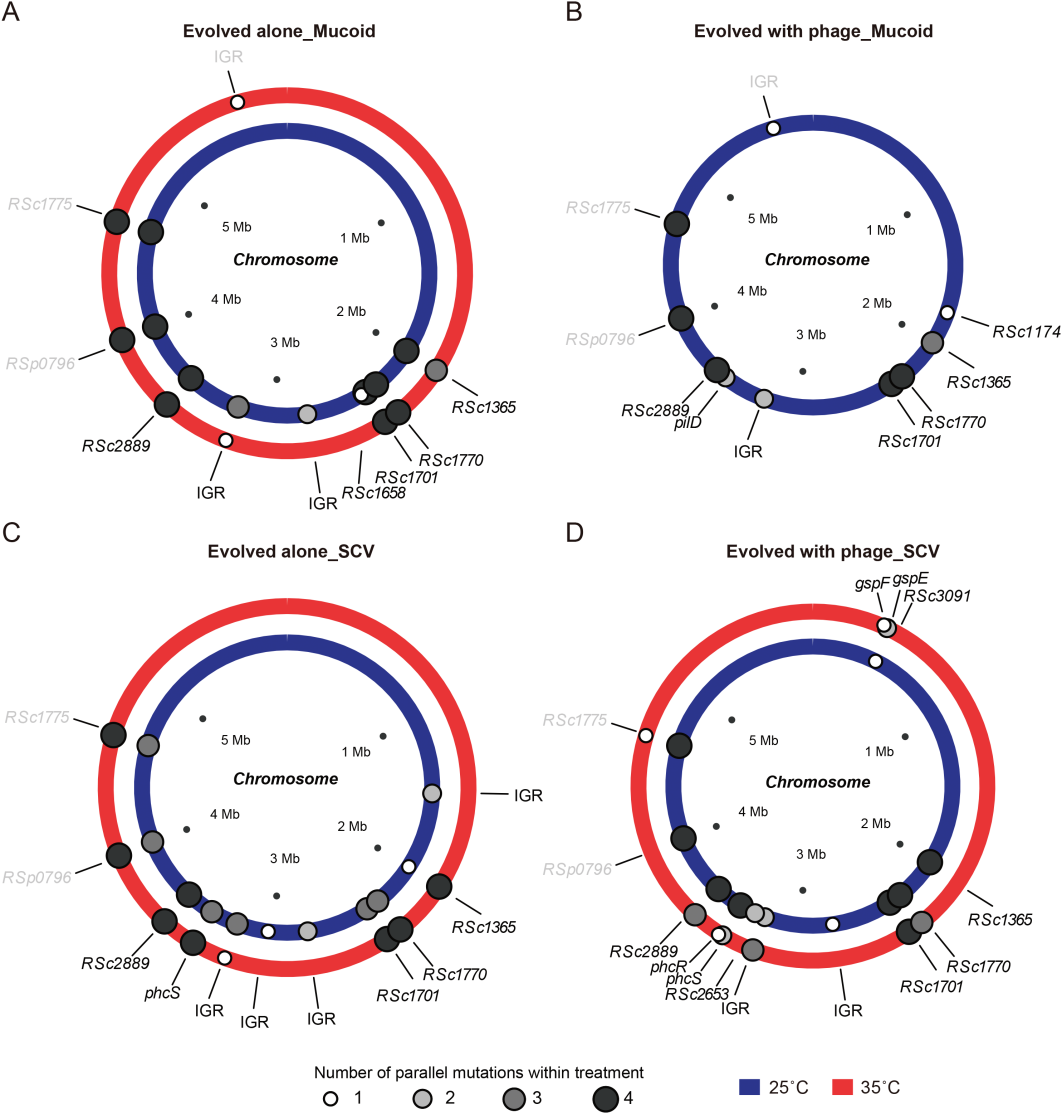
Mutations identified across different loci of clones evolved at 25 °C (blue circles) and 35 °C (red circles) in the absence (A and C) and presence of phages (B and D). (A) and (B) show mucoid and (C) and (D) SCV colony types, respectively. In all panels, the size and color of smaller dots on the circles (whole genome composed of chromosome and megaplasmid) denote for the number of parallel mutations in given loci, and black and gray text color indicate whether mutations were in the chromosome or megaplasmid, respectively. IGR denotes for mutations observed on intergenic regions (small white dots). Mutations that appeared in all clones and ancestral strain are not shown in the figure.

Most mutations were found in the chromosome and only a few mutations were found in the megaplasmid (Supplementary Table S4; *R. solanacearum* has bi-partite genome; Genin & Denny, 2012). Some mutations were observed in all treatment clones, including *RSc3188* (putative hemagglutinin-related protein), *gltB* (probable glutamate synthase [large subunit] oxidoreductase protein), and *tISRsol* (probable transposase protein), which indicates that they were potentially selected by adaptation to the NB growth media (Supplementary Table S5). Interestingly, SCVs were also associated with mutations in *phcS* quorum sensor histidine kinase gene (Table 1 and Figure 3), which activates the *phcA* virulence network through quorum-sensing signaling (Genin & Denny, 2012). Moreover, we observed parallel mutations in putative multidrug resistance protein (*RSc1365*) (Table 1 and Figure 3), which was however not clearly associated with mucoidy, prior exposure to phages or temperature environment. Interestingly, two of four mucoid phage-resistant clones that had evolved at 25 °C temperature treatment in the presence of phage had mutations in *pilD* gene (Table 1 and Figure 3), which is involved in pilus biogenesis. As these mucoid phage-resistant colony types had also considerably reduced twitching motility, it is likely that pilus was used as a receptor by the phage NNP42. To validate the role of pilus as a phage receptor, we used previously constructed *pilM* and *pilB* mutant strains for phage infections and found that both mutants were fully resistant to phage NNP42 relative to the wildtype strains (Supplementary Table S6). We also confirmed that *pilD* mutants observed in our experiment showed reduced phage adsorption, indicating that *pilD* gene was a likely phage receptor (Supplementary Figure S3).

**Table 1.** The list of observed mutations for mucoid (M) and small colony variants (SCVs) evolved in the absence and presence of phage at 25 °C and 35 °C temperature treatments. The “Type” column shows the predicted effect of given mutations, and numbers on the right show the number of replicate clones with observed mutation of all sequenced clones.

Position	Locus TAG	Gene	Type	Protein function	25 °C				35 °C		
					−P		+P		−P		+P
					M	SCV	M	SCV	M	SCV	SCV
C-2370164	RS-N_02189	*RSc1701*	Insertion	Predicted lipoprotein transmembrane	4/4	3/4	4/4	4/4	4/4	4/4	4/4
C-3598006	RS-N_03355	*RSc2889*	Insertion	Predicted transmembrane protein	4/4	4/4	4/4	4/4	4/4	4/4	3/4
C-2286347	RS-N_02118	*RSc1770*	Insertion	Predicted hydrolase/ayltransferase (Alpha/beta hydrolase superfamily)	4/4	3/4	4/4	4/4	4/4	4/4	3/4
P-911488	RS-N_04190	*RSc1775*	Insertion	Predicted hemagglutinin-related protein	4/4	3/4	4/4	4/4	4/4	4/4	1/4
P-283737	RS-N_03706	*RSp0796*	Insertion	Predicted transcription regulator protein	4/4	3/4	4/4	4/4	4/4	4/4	0/4
C-1989654	RS-N_01894	*RSc1365*	Deletion	Predicted multidrug resistance-like efflux transmembrane protein	4/4	1/4	3/4	4/4	3/4	4/4	0/4
C-3236355	Intergenic	*__*	Deletion	__	3/4	3/4	2/4	2/4	1/4	1/4	3/4
C-3415879	RS-N_03191	*phcS*	SNP/Deletion	Histidine kinase	0/4	3/4	0/4	4/4	0/4	4/4	2/4
C-2778898	Intergenic	*__*	Insertion	__	2/4	2/4	0/4	1/4	0/4	0/4	0/4
C-406950	RS-N_00359	*gspE*	SNP	Type II secretion system protein E	0/4	0/4	0/4	0/4	0/4	0/4	2/4
C-3306756	RS-N_03094	*RSc2653*	Insertion	Predicted protease signal peptide protein	0/4	0/4	0/4	2/4	0/4	0/4	0/4
C-3523802	RS-N_03289	*pilD*	Insertion	Type Ⅳ prepilin-like proteins leader peptide-processing enzyme	0/4	0/4	2/4	0/4	0/4	0/4	0/4
P-1833257	Intergenic	*__*	Deletion	__	0/4	0/4	1/4	0/4	1/4	0/4	0/4
C-432461	RS-N_00383	*RSc3091*	Deletion	Predicted outer membrane porin signal peptide protein	0/4	0/4	0/4	1/4	0/4	0/4	0/4
C-1492748	Intergenic	*__*	Complex	__	0/4	1/4	0/4	0/4	0/4	0/4	0/4
C-1492781	Intergenic	*__*	SNP	__	0/4	1/4	0/4	0/4	0/4	0/4	0/4
C-1771240	RS-N_01695	*RSc1174*	SNP	Predicted transmembrane ABC transporter protein	0/4	0/4	1/4	0/4	0/4	0/4	0/4
C-405903	RS-N_00358	*gspF*	Deletion	Predicted general secretory pathway f transmembrane protein	0/4	0/4	0/4	0/4	0/4	0/4	1/4
C-2402614	RS-N_02234	*RSc1658*	Deletion	Predicted jamm isopeptidase protein	1/4	0/4	0/4	0/4	0/4	0/4	0/4
C-3032046	Intergenic	*__*	Deletion	__	0/4	1/4	0/4	0/4	0/4	0/4	0/4
C-3417086	RS-N_03192	*phcR*	SNP	Histidine kinase	0/4	0/4	0/4	0/4	0/4	0/4	1/4

Finally, three of four sequenced small colony types that had evolved at 35 °C in the presence of phage had mutations in T2SS genes *gspF* and *gspE* (Table 1 and Figure 3), which encode secretion channel and cytoplasmic ATPase that provides the energy that drives assembly of the pseudopilus, respectively (Xavier et al., 2022). Pseudopilus is also composed of four minor pseudopilins that bear structural similarity to pilin components of type IV pilus (Hay et al., 2017) and could hence have acted as an alternative receptor for the phage NNP42. However, either of the T2SS mutants or any of the tested *phcS* mutants showed no reduction in phage adsorption (Supplementary Figure S3), which suggests that these mutations were not associated with phage binding. Together, these results suggest that phage resistance of mucoid colony types was linked to mutations in genes associated with motility, while the evolution of phage resistance and loss of virulence of SCVs was explained by mutations in quorum-sensing and T2SS-associated genes.

### Activation of restriction modification and abortive infection phage defense systems leads downregulation of potential phage receptor and virulence-associated genes

**Figure 4.**
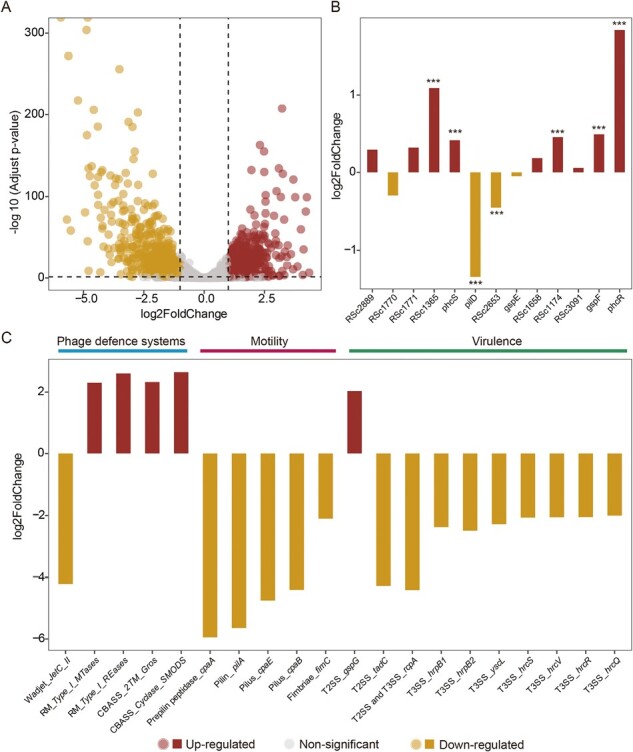
*R. solanacearum* transcriptomic analysis in the absence and presence of phage. (A) Differential expression of genes in the presence of phage with a false discovery rate < 0.05 and |log_2_FC| > 1. (B) Differential expression of genes in the presence of phage that were found to be mutated in phage treatments at the end of the selection experiment (significantly expressed genes are shown with stars with a false discovery rate < 0.05 and |log_2_FC| > 1). (C) Differential expression of genes linked to phage defense systems, motility, and virulence in the presence of phage (only genes with *p*adj < .05 and |log_2_FC| > 2 are shown). In all panels, red and yellow colors denote upregulated and downregulated genes, respectively, while gray color in (A) denotes for nonsignificance.

To study the potential role of phage defense systems for phage resistance and associated virulence trade-offs, transcriptomic analysis was conducted comparing the gene expression of ancestral *R. solanacearum* strain in the absence and presence of phage after 6-hr exposure. This time point was chosen based on one-step growth curve, showing that NNP42 densities start to increase already after 30 min of coculturing (Supplementary Figure S4), ensuring high numbers of mRNA transcripts. We found that a total 1477 of *R. solanacearum* genes were differentially expressed after phage exposure (|log_2_foldchange| > 1, FDR < 0.05, Figure 4A). Of these, around half (707) were upregulated, and another half (770) downregulated in the presence of phage. Based on KEGG pathway enrichment analysis, a total of five metabolic pathways were significantly enriched in phage compared to control treatment (*p* < .05, Supplementary Figure S5). These included propanoate metabolism, RNA degradation, synthesis and degradation of ketone bodies, bacterial secretion system, and amino acid metabolism (valine, leucine, and isoleucine degradation, Supplementary Figure S5). Interestingly, some of the genes that were mutated during the selection experiment showed clearly altered gene expression. For example, *phcR* and *phcS* virulence-associated genes that were significantly upregulated, while *pilD* and other pilus biogenesis and adherence (fimbriae) associated genes were significantly downregulated in the presence of phage (Figure 4B and C). Of 13 mutated genes that were selected for in the presence of phage, seven showed differential gene expression, indicating their role in responding to phage infection (Figure 4B). We also found that the expression of five phage defense system-related genes belonging to three different systems (Wadjet, Restriction-modification type I, and CBASS) were altered in the presence of phage (Figure 4C). Specifically, *JetC* gene belonging to Wadjet system, was downregulated, while genes linked to *MTases* and *Reases* belonging to Restriction-Modification type I system, and *SMODS* (cyclase) and *Gros* (effector) genes belonging to CBASS abortive infection system, were upregulated (Figure 4C). We also found that phage exposure led to downregulation of several virulence genes associated with motility and type II and III secretions systems (Figure 4C). Type II secretion system genes were associated with pseudopilus genes (e.g., *gspG, rcpA*) and adherence (*tadC*), while downregulated Type III secretion (T3SS) genes included several *hrp* and *hrc* genes which form the core of the T3SS, which in involved in disease development, hypersensitive reaction, and secretion of several effector proteins inside the plant (Figure 4C). Together these results suggest that already short-term exposure to phage NNP42 leads to considerable upregulation of phage defense systems and downregulation of several virulence and potential phage receptor genes in *R. solanacearum*.

## Discussion

Here, we studied how temperature affects the evolution of phage resistance and potential resistance–virulence trade-offs in *R. solanacearum*. Overall, we found that temperature had a very small effect on the evolutionary trajectory of *R. solanacearum*, even though 35 °C temperature treatment supported higher bacterial population densities in line with previous field studies (Wei et al., 2015). While the phage was effective at controlling *R. solanacearum* densities at both temperature treatments, this effect diminished in time due to phage resistance evolution. Interestingly, phage resistance was linked to the emergence of small colony variants (SCVs) that took over phage-exposed populations. SCVs also increased in high frequency in the absence of phages likely due to their relatively higher growth rate compared to ancestral bacterium. At the molecular level, phage resistance was linked to loss of quorum sensing and defects in genes associated with pilus biosynthesis and T2SS, which could explain clear trade-offs between phage resistance and virulence in planta. Moreover, a similar trade-off was found between the activation of phage defense systems and expression of motility and virulence-associated genes in the absence of phage resistance evolution. While more work is required to compare the rate, dynamics, and measurements of temperature dependency (we used only one measurement temperature) of phage resistance evolution, our results suggest that resistance–virulence trade-offs are pervasive with *R. solanacearum* and not affected by the environmental temperature.

Phage resistance evolved readily in both temperature treatments during the selection experiment. Mechanistically, phage resistance was linked to SCVs that had mutations in *phcS* gene, which is a sensory kinase of two-component system responsible for the activation of *R. solanacearum* primary virulence network *phcA* in response to quorum-sensing autoinducers (Genin & Denny, 2012; Kai, 2023). Interestingly, phage-resistant SCVs were also selected in the absence of phages, and likely reason for this was that *phcS* mutations increased the growth rate of SCVs, making them more competitive relative to the ancestral colony types. Mutations in *phcS* often lead to a lack or reduced of *phcA* expression, improving the growth of *R. solanacearum* due to a lack of costly virulence factor production (Clough et al., 1997; Kai, 2023). However, this growth difference was not enough to lead to a complete selective sweep of SCVs in the absence of phages. In contrast, SCVs dominated phage treatments except for one replicate at 25 °C treatment where also mucoid colony types persisted. While we found that SCVs were stable phenotypes after a few rounds of growth on agar plates, their long-term stability and potential reversion to mucoid types should be tested in the future. Previous studies have also found that changes in *R. solanacearum* colony morphology are associated with fitness benefits in specific niches (Perrier et al., 2019). For example, mutations in *efpR* gene have been associated with stable maintenance of two colony types that are specialized regarding their carbon metabolism and growth in planta (Guidot et al., 2014; Perrier et al., 2016, 2019). Previous studies on using temperate phages of *Ralstonia* characterized by chronic infection have also shown that phage-infected cells have small colony morphology and reduced expression of *phcA* gene, suggesting that resistance to both lytic and temperate phages is associated with QS-signaling and small colony types (Addy et al., 2012). In a few cases, SCVs were not associated mutations in *phcS* gene, suggesting that small colony morphology is also governed by other genes. Together with previous studies, our results suggest that small colony morphology could be a reliable marker for genetic adaptation in *R. solanacearum*.

What were the molecular and genetic bases of phage resistance? While the receptor of NNP42 phage is not yet known, our sequencing results suggest a few potential candidate genes. First, we observed an alternative phage resistance trajectory at 25 °C-phage treatment in one of the replicate populations. Here, phage-resistant colonies showed mucoid colony type that had clearly reduced twitching motility. At the genetic level, this change could be explained by mutations in *pilD* gene, which is prepilin peptidase that processes prepilins before they are added to the type IV pilus (T4P) (Xavier et al., 2022). T4P is a common phage receptor with various bacteria (Bondy-Denomy et al., 2016; James et al., 2012) and has previously been linked to phage resistance also in *R. solanacearum* (Narulita et al., 2016; Xavier et al., 2022). In support of this, we found that *pilD* mutants showed a significant reduction in phage adsorption and that genetically engineered *pilM* and *pilB* mutant strains were resistant to NNP42 phage relative to otherwise isogenic ancestral strains. Moreover, our transcriptomic analysis showed that already short-term exposure of ancestral *R. solanacearum* to the NNP42 phage led to a clear downregulation of *pilD* gene, which encoded for a prepilin peptidase that has been shown to act in both T4P and T2SS and has been previously associated to phage resistance in another podophage that was still able to adsorb to the phage-resistant mutants (Xavier et al., 2022).

We also found parallel mutations in *gspF* and *gspE* genes encoding T2SS secretion channel and cytoplasmic ATPase that provides the energy that drives the assembly of the pseudopilus (Xavier et al., 2022). One potential scenario is that NNP42 phage can bind either T4P or T2SS because these systems share some similar structural components: type II secretions system encodes pseudopilus, which is composed of four minor pseudopilins that bear structural similarity to pilin components of type IV pilus (Hay et al., 2017). However, we found no significant reduction in phage adsorption by T2SS mutants, which suggest that *gspF* and *gspE* genes constrained phage infections elsewhere downstream of host binding as observed previously by Xavier et al. (2022). For example, Xavier et al. (2022) recently showed that overexpression of *gspG* could lead to phage resistance through the expression of hyperpseudopilus, which could also alter the functionality of the T2SS. While our findings are similar to a previous study (Xavier et al., 2022), more work is needed to understand whether observed phage resistance–virulence trade-offs rise generally across genetically diverse RSSC. Interestingly, while no mean difference in the level of phage resistance was observed between the two temperature environments, *pilD* mutants evolved only at 25 °C and T2SS mutants only at 35 °C temperature treatments. While the number of these mutants and treatment replicates is small, this suggests that environmental temperature could potentially shape the genetic trajectory of phage resistance.

How did the loss of quorum-sensing signaling then modulate phage resistance in *R. solanacearum*? None of the SCVs had mutations in pilus biosynthesis genes and showed no change in phage adsorption. However, it is known that quorum-sensing system is required for the activation of *phcA*, which in turn controls expression and secretion of plant cell wall degrading enzymes through T2SS (Genin & Denny, 2012). The loss of quorum sensing could have hence led to reduced T2SS activity, making SCVs resistant to phages. This does not however explain why phages could not infect *R. solanacearum* cells using T4P and why T4P mutants were not selected in the SCV background. One explanation could be that the experimental growth conditions and temperature affected the levels of T4P expression as shown previously (Bocsanczy et al., 2012, 2014). Moreover, while T4P could have risen transiently, they might have been outcompeted by *phcS* mutants that showed an increase in their growth rate. To fully answer these questions, more studies are needed in the future.

We also found that short-term phage exposure led to increased expression of two phage defense systems—Restriction Modification (RM) type I and CBASS abortive infection system—with the phage susceptible ancestral *R. solanacearum* strain. RM type I is common for *R. solanacearum* strains (Castillo et al., 2020) and helps bacteria to recognize and destroy foreign DNA, which has not been methylated. Instead, CBASS trigger altruistic cell suicide upon phage infection through the activation of cyclase and effectors genes (Duncan-Lowey & Kranzusch, 2022). While it has previously shown that different phage defense systems can work synergistically providing enhanced resistance against phages (Tesson & Bernheim, 2023; Wu et al., 2022), it is also possible that these systems were expressed sequentially in our experiment. For example, CBASS could be activated later in response to phage RNA structures, while RM will be active much earlier as it is activated by the entry of phage DNA into the host cell (Enikeeva et al., 2010; Tesson & Bernheim, 2023). However, temporal transcriptomics is required to validate this hypothesis of potential multi-layered defence. Moreover, we observed reduced expression of Wadjet system, which is a recently discovered anti-plasmid system that identifies invading DNA by sensing its topology and subsequently cleaving closed-circular DNA (Deep et al., 2022). This system may also serve as protection against uncontrolled natural transformation or, conversely, it might have a specific role in targeting single-stranded DNA phages (Doron et al., 2018). While it has been observed across *R. solanacearum* species complex in relatively small frequency (Castillo et al., 2020), our data suggest it is actively responding to phage infections. Unfortunately, we were not able to assess the activity of defense systems in our evolved phage-resistant clones. One potential future experiment will be the comparison of defense system expression between ancestral and SCV colony types in different receptor mutation backgrounds. Moreover, in the future, it would be interesting to quantify defense system expression in response to phages at different temperatures and to test whether coevolved phage mutants emerge over longer time scale.

In general, phage resistance was costly, leading to reduction in *R. solanacearum* carrying capacity and loss of virulence *in planta*. Our findings are similar to several previous studies where defects in *R. solanacearum* quorum-sensing signaling (Hikichi et al., 2017; Kelman, 1954), T2SS (Xavier et al., 2022), and pilus biosynthesis (Corral et al., 2020; Liu et al., 2001; Persat et al., 2015) have led to a reduction in virulence. In our experiments using direct stem injection, all mutants showed complete loss of virulence, indicative of similarly high costs. Interestingly, the upregulation of phage defense systems was accompanied by downregulation of pilus and type II secretions systems genes that were associated to phage resistance in the selection experiment. Moreover, a clear reduction in the expression of several virulence genes associated with-type III secretion system was observed upon short-term phage exposure. The type III secretion system is important for the successful host colonization and evasion of plant immune defenses via secretion of effector proteins (Genin & Denny, 2012). Our results hence demonstrate that activation of phage defense systems could potentially constrain plant colonization through trade-offs with virulence gene expression even in the absence of genetic phage resistance—a hypothesis that needs to be tested in planta in the future.

In conclusion, our results suggest that *R. solanacearum* can defend itself against NNP42 phage by activating specific phage defense systems or by evolving fully resistant through selection for phage receptor mutations. In both scenarios, increased phage resistance came with a cost of reduced virulence, and overall, temperature had no effects on the evolution of phage resistance–virulence trade-offs. In the biocontrol perspective, our results suggest that phages could be used to control *R. solanacearum*. Even with an increase in the frequency of phage-resistant mutants, these mutants were less virulent, which could constrain bacterial wilt disease outbreaks. While it remains to be explored if similar evolutionary trajectories will arise in more complex rhizosphere microbiomes, previous studies suggest that phage resistance evolution in the tomato rhizosphere can reduce *R. solanacearum* competitive ability (Wang et al., 2019). Evolutionary steering of pathogen virulence with phages could hence offer a novel way to improve the efficacy of phage biocontrol applications.

## Supplementary Material

qrad056_suppl_Supplementary_Tables_S1-S6_Figures_S1-S5

## Data Availability

The datasets and code generated during the current study are available in the dryad digital repository, https://doi.org/10.5061/dryad.tdz08kq5d. The sequences of evolved *R. solanacearum* strains and transcriptomics raw data were deposited into the NCBI Sequence Read Archive (SRA) database (Accession Numbers: PRJNA1028400, PRJNA1032726).
