# Supplementary material for: Phage selection drives resistance–virulence trade-offs in Ralstonia solanacearum plant-pathogenic bacterium irrespective of the growth temperature
Source: Evol Lett. 2023 Nov 11;8(2):253–66. doi: 10.1093/evlett/qrad056 (PMC10959482; doi:10.1093/evlett/qrad056)
Supplement: qrad056_suppl_Supplementary_Tables_S1-S6_Figures_S1-S5 [file qrad056_suppl_supplementary_tables_s1-s6_figures_s1-s5.pdf]

Phage selection drives resistance-virulence trade-offs in *Ralstonia solanacearum* plant pathogenic bacterium irrespective of the growth temperature

Jianing Wang[1,#], Xiaofang Wang[1,#,*], Keming Yang[1], Chunxia Lu[1], Bryden Fields[2], Yangchun Xu[1], Qirong Shen[1], Zhong Wei[1], Ville -Petri Friman[1,2,3]

[1] Key Lab of Organic-based Fertilizers of China and Jiangsu Provincial Key Lab of Solid Organic Waste Utilization, Nanjing Agricultural University, Nanjing 210095, P.R. China

[2]Department of Microbiology, University of Helsinki, 00014, Helsinki, Finland.

[3]University of York, Department of Biology, Wentworth Way, York, YO10 5DD, UK

[#]These authors contribute equally to this work

*Correspondence: wangxf@njau.edu.cn (Xiaofang Wang)

Supplementary figures 1-5

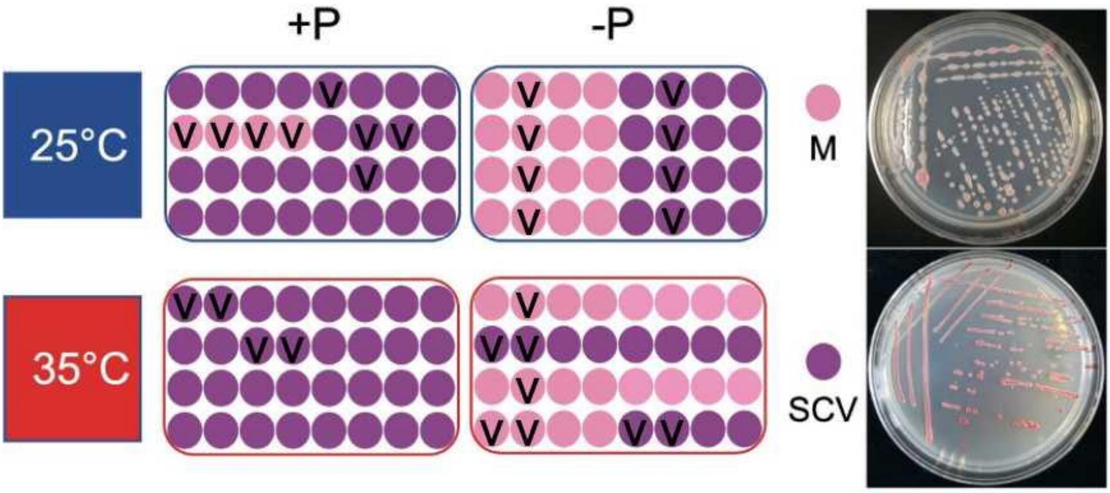

Figure S1. The selection of 32 colonies from each treatment for quantifying evolutionary changes at the end of the selection experiment. Four replicates per each treatment (columns with each box) were used to isolate 8 colonies (rows on each box) per treatment replicate. If given treatment contained only one colony type, 8 colonies of this one type were randomly isolated. If treatment contained two colony types, four of each were isolated in random. Pink and purple dots represent mucoid and SCV *R. solanacearum* colony types. All isolated clones were used to test the resistance to phage and growth traits (including maximum growth rate and carrying capacity), and clones with

“ v ” mark were used for motility, biofilm formation, virulence *in planta* assays and genetic assays.

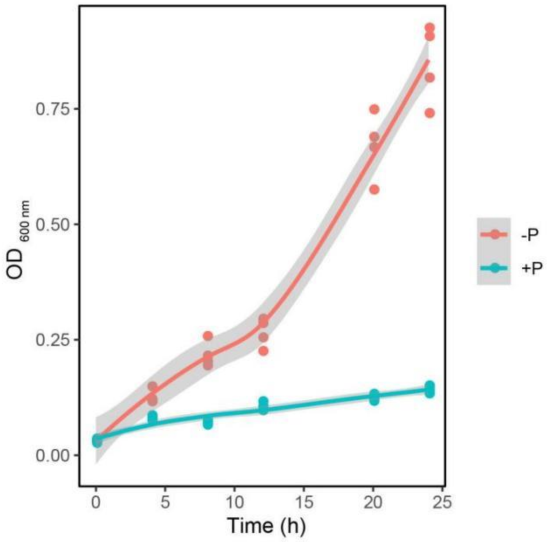

Figure S2. Population density dynamics of the ancestral *R. solanacearum* strain in the presence (+P) and absence of ancestral phage (-P) was used as control treatment. The experiment was conducted on 96-well microplates at 30 °C with shaking at 170 rpm and bacterial growth was measured at 0, 4 h, 8 h, 12 h, 18 h and 24 h after initial inoculation. The MOI of phage treatment was 0.1.

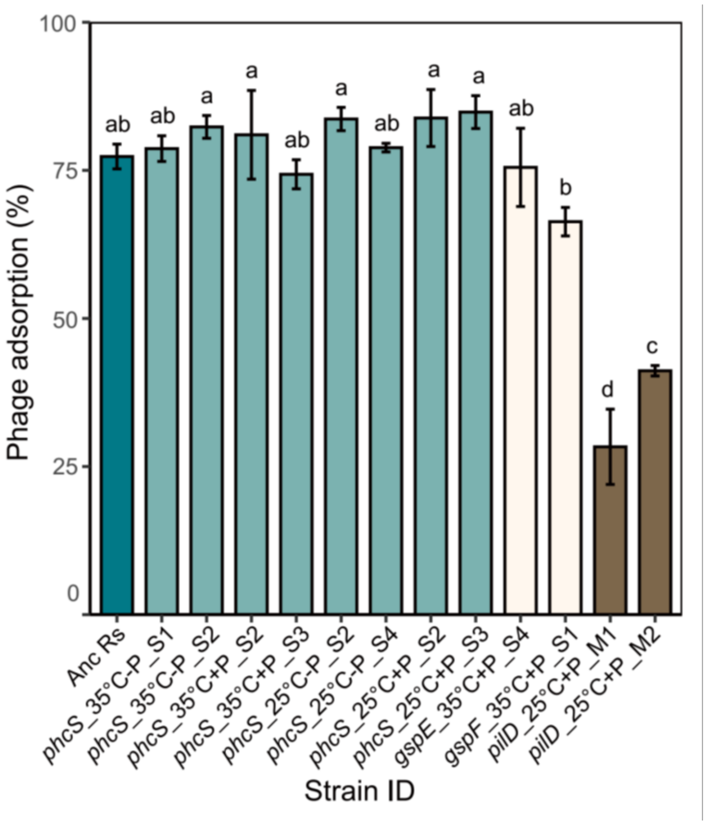

Figure S3. Adsorption of ancestral NNP42 phage to ancestral RS-N and *phcS*, *gspE*, *gspF* and *pilD* mutants isolated at the end of the selection experiment. Different lowercase letters above barplots denote significant differences between treatments ($P < 0.05$). Adsorption proportion of phage were conducted using a modification of the method by (Denes et al. 2015). Briefly, bacteria and phage mixtures (MOI=0.1) were incubated for 30 min at 30°C. Subsequently, the mixture was centrifuged at 16000 ×g for 3 min. The supernatants were then filtered through 0.22 μm-pore size surfactant-free cellulose acetate syringe filters. The percentage adsorption was defined as the loss of phages (percent) from each sample after coincubation with bacteria relative to the initial phage density.

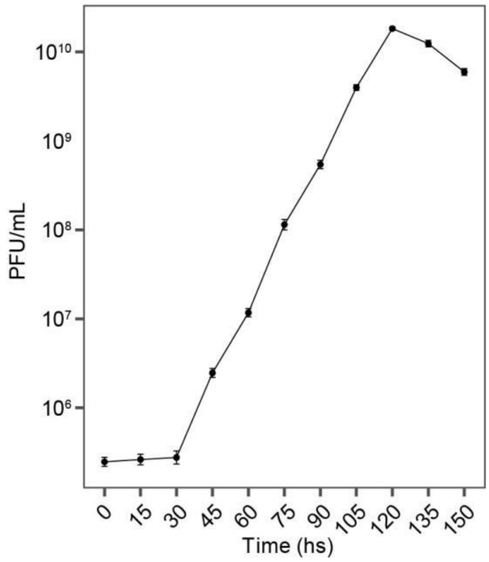

Figure S4. One-step growth curve of NNP42 phage infecting ancestral *R. solanacearum* strain. Briefly, 2 mL of RS-N culture in its exponential growth phase was infected with 20 μL of phage lysate (MOI = 0.1) 30 °C. Phage replication was quantified during 15-minute temporal sampling (see X-axis) where collected samples were centrifuged at 6000 × g for 3 min, and phage titers determined using the plague assay.

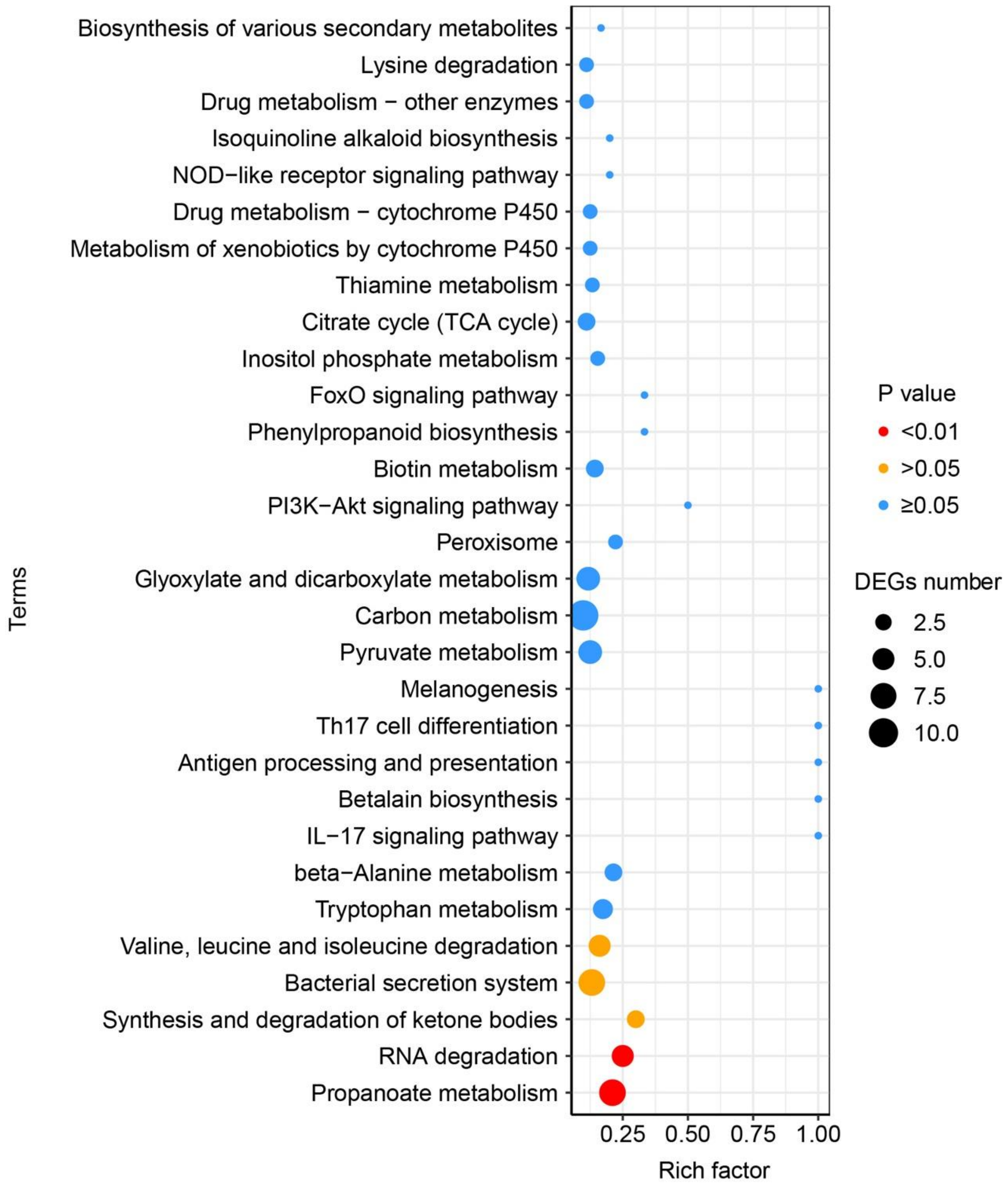

Figure S5. KEGG enrichment analysis for differentially expressed genes in the presence of phage. The X-axis represents the enrichment factor, which is the ratio of the number of differentially expressed genes in the given pathway (Y-axis) relative to the number of genes annotated in this pathway. A larger value (reflected as dot size) indicates a greater degree of enrichment. Y-axis shows the metabolic pathway with top 30 enrichment factors. The significances are shown with dot colors.

Supplementary tables 1-6

Table S1. Statistical analyses regarding *R. solanacearum* and phage population density dynamics. (a) Three-way repeated measures ANOVA explaining variation in *R. solanacearum* densities by temperature and phage treatments for 5 transfers. (b) Two-way repeated measures ANOVA explaining variation in phage densities by temperature for 5 transfers. Significant results are bolded, and significance levels are shown on stars where *$P < 0.05$, **$P < 0.01$ and ***$P < 0.001$.

Source of variation	Df	Sum of squares within groups	Mean squares within groups	F	P
(a) *R. solanacearum* densities					
Error: factor (ID)					
Temperature	1	5.681	5.681	73.989	**2.96E-10*** **
Phage	1	3.747	3.737	48.800	**3.43E-08*** **
Temperature × phage	1	0.014	0.014	0.182	0.673
Residuals	36	2.764	0.077		
Error: Within					
Time	5	0.740	0.148	7.316	**2.90E-06*** **
Temperature × time	5	0.289	0.057	2.855	**0.0166***
Phage × time	5	0.943	0.18857	9.327	**6.45E-08*** **
Temperature × phage × time	5	0.391	0.07824	3.870	**0.002** **
Residuals	180	3.639	0.02022		
(b) Phage densities					
Error: factor (ID)					
Temperature	1	28.61	28.612	32.75	**2E-05*** **
Residuals	18	15.73	0.874		
Error: Within					
Time	2	84.88	42.44	86.963	**1.65E-14*** **
Temperature × time	2	0.38	0.19	0.386	0.683
Residuals	36	17.57	0.49		

Table S2. Statistical analysis explaining variation in *R. solanacearum* colony type frequencies by temperature, phage or their interaction based on Two-way ANOVA at the last time point of the selection experiment. Significant results are bolded, and significance levels are shown on stars where *$P < 0.05$, **$P < 0.01$ and ***$P < 0.001$.

Source of variation	Df	Sum of squares	Mean square	F	P
Temperature	1	0.155	0.155	2.218	0.162
Phage	1	0.491	0.491	7.016	**0.021***
Temperature × phage	1	0.159	0.159	2.273	0.157
Residuals	12	0.840	0.700		

Table S3. Statistical analysis explaining variation in *R. solanacearum* phage resistance (a), maximum growth rate (b), maximum density (c), twitching motility (d), biofilm formation (e) and virulence (d) by temperature, phage or their interaction based on Two-way ANOVA at the last time point of the selection experiment. Significant results are bolded, and significance levels are shown on stars where **P* < 0.05, ***P* < 0.01 and ****P* < 0.001.

**Source of variation**	**Df**	**Sum of squares**	**Mean square**	**F**	**P**
(a) Resistance to ancestral phage					
Temperature	1	0.018	0.018	6.459	**0.023***
Phage	1	0.800	0.800	280.752	**4.030E-11*****
Colony type	1	1.268	1.268	445.207	**1.451E-12*****
Temperature × phage	1	0.006	0.006	2.099	0.168
Temperature × colony type	1	0.019	0.019	6.516	**0.022***
Phage × colony type	1	0.245	0.245	86.063	**1.329E-07*****
Residuals	15	0.043	0.003		
(b) Maximum growth rate					
Temperature	1	1.968E-04	1.968E-04	26.621	**1.164E-04*****
Phage	1	2.384E-05	2.384E-05	3.226	0.093
Colony type	1	2.173E-04	2.173E-04	29.393	**7.075E-05*****
Temperature × phage	1	3.223E-05	3.223E-05	4.360	0.054
Temperature × colony type	1	1.300E-08	1.300E-08	0.002	0.967
Phage × colony type	1	2.142E-05	2.142E-05	2.898	0.109
Residuals	15	1.109E-04	7.393E-06		
(c) Maximum density					
Temperature	1	0.010	0.010	4.666	**0.047***
Phage	1	0.042	0.042	19.598	**4.897E-04*****
Colony type	1	0.189	0.189	87.965	**1.154E-07*****
Temperature × phage	1	0.007	0.007	3.268	0.091
Temperature × colony type	1	0.007	0.007	3.222	0.093
Phage × colony type	1	0.006	0.006	2.900	0.109
Residuals	15	0.032	0.002		
(d) Twitching motility					
Temperature	1	3.857	3.857	2.264	0.147
Phage	1	0.210	0.210	0.123	0.729
Colony type	1	14.510	14.510	8.516	**0.008****
Temperature × phage	1	0.604	0.604	0.355	0.558
Temperature × colony type	1	0.081	0.081	0.047	0.839
Phage × colony type	1	27.040	27.040	15.870	**0.000*****
Residuals	21	35.780	1.704		
(e) Biofilm formation					

Temperature	1	0.346	0.346	1.806	0.193
Phage	1	0.000	0.000	0.000	0.993
Colony type	1	8.555	8.555	42.370	**1.895E-06*****
Temperature × phage	1	0.053	0.053	0.264	0.613
Temperature × colony type	1	0.020	0.020	0.099	0.756
Phage × colony type	1	0.490	0.490	2.426	0.134
Residuals	21	4.240	0.202		
(f) Disease incidence					
Temperature	1	0.0203	0.0203	1.987	0.173
Phage	1	0.207	0.207	20.310	**1.935E-04*****
Colony type	1	0.341	0.341	33.413	**9.745E-06*****
Temperature × phage	1	0.009	0.009	0.910	0.351
Temperature × colony type	1	0.079	0.079	7.714	**0.011***
Phage × colony type	1	0.102	0.102	9.992	**0.004****
Residuals	21	0.214	0.0102		

Table S4. Total number of mutations in evolved sequenced colonies in different treatments.

Treatment	Location	Deletions	Insertions	SNPs	Total
25°C-P-M	Chromosome	8	34	4	46
25°C-P-M	Megaplasmid	0	24	0	24
35°C-P-M	Chromosome	4	32	4	40
35°C-P-M	Megaplasmid	1	24	0	25
25°C+P-M	Chromosome	5	34	5	44
25°C+P-M	Megaplasmid	1	24	0	25
25°C-P-NM	Chromosome	8	32	5	45
25°C-P-NM	Megaplasmid	0	22	0	22
35°C-P-NM	Chromosome	8	32	5	45
35°C-P-NM	Megaplasmid	0	24	0	24
25°C+P-NM	Chromosome	11	35	4	50
25°C+P-NM	Megaplasmid	0	24	0	24
35°C+P-NM	Chromosome	6	30	7	43
35°C+P-NM	Megaplasmid	0	17	0	17

Table S5. The list of mutations common for all treatments that likely evolved in response to NB or other experimental conditions. The 'Type' column shows the predicted effect of given mutations, and numbers on the right show the number of replicate clones with observed mutation of all sequenced clones in that treatment.

					25 °C				35 °C		
**Position**	**Locus TAG**	**Gene**	**Type**	**Protein function**	**-P**		**+P**		**-P**		**+P**
					**M**	**SCV**	**M**	**SCV**	**M**	**SCV**	**SCV**
C-316392	RS-N_00287	*RSc3188*	Insertion	Putative hemagglutinin-related protein	4/4	4/4	4/4	4/4	4/4	4/4	4/4
C-370410	Intergenic	__	Insertion	__	4/4	4/4	4/4	4/4	4/4	4/4	4/4

C-774686	Intergenic	__	Insertion	__	4/4	4/4	4/4	4/4	4/4	4/4	4/4
C-2144146	Intergenic	__	SNP	__	4/4	4/4	4/4	4/4	4/4	4/4	4/4
C-3448123	Intergenic	__	Insertion	__	4/4	4/4	4/4	4/4	4/4	4/4	4/4
C-3679765	RS-N_03435	*gltB*	Insertion	Predicted glutamate synthase (Large subunit) oxidoreductase protein	4/4	4/4	4/4	4/4	4/4	4/4	4/4
P-4703	RS-N_03478	*tISRsol*	Insertion	Predicted transposase protein	4/4	4/4	4/4	4/4	4/4	4/4	4/4
P-1146789	Intergenic	__	Insertion	__	4/4	4/4	4/4	4/4	4/4	4/4	4/4
P-2034318	Intergenic	__	Insertion	__	4/4	4/4	4/4	4/4	4/4	4/4	4/4

Table S6. Phage resistance of *pilB*, *pilM* mutants and their isogenic wildtype strains. Phage resistance was measured as bacterial growth in the presence of phage in liquid NB media and values in the table show OD values at 600 nm. Mutants with in-frame deletion of target genes were generated with pK18mobsacB-based homologous recombination as described previously (Zhang et al. 2015). Briefly, the DNA fragments flanking target genes was conjugated with joint PCR and cloned into pK18mobsacB to generate pK18pilM which was subjected to in-frame deletion of genes *pilM*. After validating sequences, the plasmid was transferred into *R. solanacearum* by conjugation with S17-1 (lab strain), and *pilM* mutant was generated and confirmed by colony PCR with primer pairs (Wang et al. 2023).

Strains	Growth in liquid in the presence of phage	Growth in liquid in the absence of phage
OE1-1 (wildtype of *pilB* mutant)	0.348	1.377
QL-Rs1115 (wildtype of *pilM* mutant)	0.526	1.639
PilB mutant	1.298	1.406
PilM mutant	1.768	1.756

References:

Denes, T., H. C. den Bakker, J. I. Tokman, C. Guldimann, and M. Wiedmann. 2015. Selection and Characterization of Phage-Resistant Mutant Strains of

Reveal Host Genes Linked to Phage Adsorption. Appl. Environ. Microbiol. 81:4295-4305.

Wang, J. N., W. Raza, G. F. Jiang, Z. Yi, B. Fields, S. Greenrod, V. P. Friman, A. Jousset, Q. R. Shen, and Z. Wei. 2023. Bacterial volatile organic compounds attenuate pathogen virulence via evolutionary trade-offs. ISME J. 17:443-452.

Zhang, Y., F. Luo, D. S. Wu, Y. Hikichi, A. Kiba, Y. Igarashi, W. Ding, and K. Ohnishi. 2015. PrhN, a putative marR family transcriptional regulator, is involved in positive regulation of type III secretion system and full virulence of Ralstonia solanacearum. Front. Microbiol. 6.
